# Landscape of the RBD-specific IgG, IgM, and IgA responses triggered by the inactivated virus vaccine against the Omicron variant

**DOI:** 10.1038/s41421-022-00380-8

**Published:** 2022-02-15

**Authors:** Jun-biao Xue, Dan-yun Lai, He-wei Jiang, Huan Qi, Shu-juan Guo, Yuan-shou Zhu, Hong Xu, Jie Zhou, Sheng-ce Tao

**Affiliations:** 1 Key Laboratory of Systems Biomedicine (Ministry of Education), Shanghai Center for Systems Biomedicine, Shanghai Jiao Tong University, Shanghai, China; 2 School of Biomedical Engineering, Shanghai Jiao Tong University, Shanghai, China; 3 Foshan Fourth People’s Hospital, Foshan, Guangdong China

**Keywords:** Immunology, Biological techniques

Dear Editor,

The Omicron variant (B.1.1.529) possesses ~32 mutations in its spike protein, and at least 15 amino acid changes in the receptor-binding domain (RBD)^1^. The Omicron variant is worsening the COVID-19 pandemic largely due to the significant escape from the neutralization antibodies^2^. To combat the Omicron variant, one of the most effective methods is vaccination, including boosters. In China and some other countries, the dominant form of vaccine is derived from inactivated virus^3^. Further, in China, the vaccination campaign was carried out sequentially, starting from at-risk groups and big cities, and then on to the general public and smaller cities. Thus, across the country, there are many people at each of the different vaccination stages, namely unvaccinated, the 1st dose, the 2nd dose, and the 3rd (booster) dose. It is of great interest to know what level of protection that the inactivated virus vaccine can provide against the Omicron variant at each of these vaccination stages for rational optimization of vaccination strategies.

Here we report the first landscape of the vaccine-stimulated RBD-specific antibody responses (IgG, IgA, and IgM) against the Omicron variant. We generated the map using a protein microarray, by analyzing longitudinal sera collected over 1 year from individuals immunized with 3 doses of an inactivated virus vaccine. The IgG response to RBD-Omicron is 1/3–1/5 that to RBD-wild type (WT); ~6× higher after the booster dose versus the 2nd dose; and reaches a plateau in ~2 weeks after the booster dose, then drops ~5× after another 2 weeks. Similar results were obtained for IgA and IgM.

It is well known that RBD-specific IgG responses are highly correlated with their neutralization activity against the authentic SARS-CoV-2 virus^4^. To enable a rapid and systematic evaluation of the immuno-neutralization activity against the Omicron variant, we constructed a protein microarray including both the WT and the Omicron RBDs (Supplementary Fig. S1a, b). We verified the microarray with 12 SARS-CoV-2 RBD-specific monoclonal antibodies (Supplementary Fig. S1c) and 14 convalescent sera (Supplementary Table S1, cohort 1; Fig. S1d). We clearly demonstrated that the Omicron variant RBD binds much weaker to a range of neutralizing antibodies, likely indicating an increased ability of this variant to escape their protection^2^. For convalescent sera, we also found lower binding to the Omicron RBD, and thus even patients who have recovered from a previous infection are still in danger. These results support the recent conclusion that many of the current mAbs do not provide adequate neutralization to the Omicron variant^5^, and the protection from the previous WT virus infection against the Omicron variant needs to be strengthened^6^.

Throughout 2021, we collected sera from a cohort of 13 individuals (Supplementary Table S1, cohort 2) who have now been fully vaccinated (the 1st dose, the 2nd dose, and the booster dose) with the inactivated virus vaccine BBIBP-CorV at 16 time points (Fig. 1a).

**Fig. 1:**
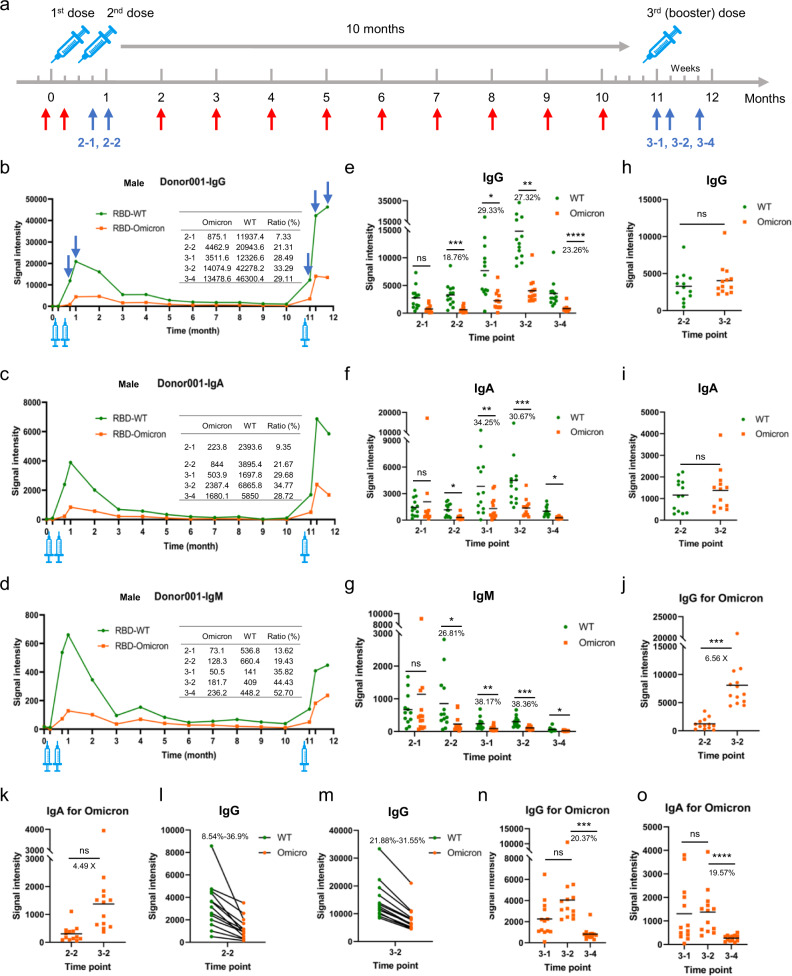
RBD-specific antibody responses of longitudinal sera collected from vaccinated individuals. **a** Scheme of immunization and sampling of the longitudinal cohort. Vertical arrows indicate the 16 time points for collecting sera. **b**–**d** The trends of RBD-specific antibody responses (IgG (**b**), IgA (**c**), and IgM (**d**)) during 1 year of a representative individual (Donor001) that was immunized with three doses of inactivated virus vaccine. **e**–**g** Antibody responses (IgG (**e**), IgA (**f**), and IgM (**g**)) against RBD-WT and RBD-Omicron at 5 time points with relatively higher antibody levels, i.e., 2-1 and 2-2 represent 1 and 2 weeks after the 2nd dose, respectively; 3-1, 3-2, and 3-4 represent 1, 2, and 4 weeks after the 3rd (booster) dose, respectively. **h**, **i** The IgG (**h**) and IgA (**i**) responses against RBD-Omicron at 3-2 and against RBD-WT at 2-2. **j**, **k** The IgG (**j**) and IgA (**k**) responses against RBD-Omicron at 2-2 and 3-2. **l**, **m** The IgG responses between RBD-WT and RBD-Omicron at 2-2 (**l**) and 3-2 (**m**). **n**, **o** The trends of RBD-Omicron specific IgG (**n**) and IgA (**o**) responses after the 3rd dose.

To determine the extent to which the IgG responses to this vaccine can provide neutralization against the Omicron variant at different stages of vaccination, we examined serum samples of cohort 2 with the microarray. Mucosal immunity is a key component of our immune response to SARS-CoV-2 infection, and IgA plays a major role in this mucosal immunity^7,8^. IgM level is important for immune protection. Thus, we also examined the IgA and IgM responses of cohort 2. We first analyzed the full set of samples (16 time points) of four individuals (Donor001–Donor004). From the 1st to the 16th time points, similar trends of IgG (Fig. 1b and Supplementary Fig. S2a–c), IgA (Fig. 1c and Supplementary Fig. S3a–c), and IgM (Fig. 1d and Supplementary Fig. S4a–c) responses were observed for all the four individuals. We found that the antibody responses were similar between RBD-WT and RBD-Omicron: The antibody responses were barely detectable 1 week after the 1st dose; a clear response was observed 1 week after the 2nd dose, maintained for 2 weeks after the 2nd dose, and then gradually decreased until the booster dose. After the booster dose, the antibody responses sharply increased and reached a plateau after 2–4 weeks. Overall, the IgG and IgA responses of the booster dose are much greater than those of the 2nd dose (Fig. 1b, c), whereas the IgM response of the booster dose is similar to or slightly lower than that of the 2nd dose (Fig. 1d). For all the 16 time points, the antibody responses against RBD-Omicron are significantly lower than those against RBD-WT.

To further illustrate the differences in the antibody responses to these RBDs, the antibody responses at 5 time points with relatively higher antibody levels were analyzed for all the 13 individuals (namely, 2-1, 2-2, 3-1, 3-2, and 3-4). Except 2-1, the averaged IgG responses to RBD-Omicron ranged from 18.76% to 29.33% of those to RBD-WT (Fig. 1e). Quantitatively, the averaged IgA responses to the RBD-Omicron were ~30% of those to RBD-WT (Fig. 1f). The averaged IgM responses, except 2-1, ranged from 26.81% to 38.36% of those to RBD-WT (Fig. 1g). All these differences were statistically significant.

A particularly noteworthy aspect of this analysis is that it provides an indication of the extent to which the booster can provide protection against the Omicron variant. We found that, in general, the highest IgG responses to RBD-Omicron after the booster dose (3-2) were comparable to the highest responses to RBD-WT after the 2nd dose (2-2) (Fig. 1h). Similar result was observed for IgA (Fig. 1i). Overall, the IgG and IgA responses to RBD-Omicron at 3-2 to those at 2-2 are 6.56× and 4.49×, respectively (Fig. 1j, k). These results indicate that to combat the Omicron variant, the booster dose is necessary, and could provide comparable protection against the Omicron variant to that of the 2nd dose against the WT strain.

As inferred from the full-range time point analysis (Fig. 1b–d), inspection of the responses at 2-2 and 3-2 confirmed the overall lower responses to RBD-Omicron than to RBD-WT for IgG (Fig. 1l, m), IgA (Supplementary Fig. S3d, e) and IgM (Supplementary Fig. S4d, e). Further, we also found that the RBD-Omicron responses after the booster gradually decreased after a plateau, with average signals of only 20.37% and 19.57% at 3-4 compared to those at 3-2 for IgG (Fig. 1n) and IgA (Fig. 1o), respectively. Thus, this confirms a similar decrease in IgG and IgA responses as observed with the WT strain, and indicates a decrease in protection against the Omicron variant in two weeks.

To further illustrate the results of Fig. 1g, we compared the IgM responses to RBD-WT at 2-2 and 3-2, RBD-Omicron at 2-2 and 3-2. The IgM responses were averagely lower at 3-2 for both RBD-WT (Supplementary Fig. S4f) and RBD-Omicron (Supplementary Fig. S4g). Further, in agreement with the results of the IgG responses, the average IgM signal decreased at 3-4 to a value that is only 19.69% of that at 3-2 (Supplementary Fig. S4h), indicating a decrease of IgM against Omicron variant in two weeks.

According to humoral immunity, IgG, IgA, and IgM all can provide protection against SARS-CoV-2. However, there is still no systematical comparisons among these three types of antibodies. Are these antibodies complementary to each other at different vaccination stages? To answer this, we first analyzed the correlations between WT and Omicron for Donor001–Donor004. High correlations were obtained for all these analyses. Specifically, for Donor001–Donor004, the IgG correlations are 0.979, 0.994, 0.984, and 0.999, the IgA correlations are 0.968, 0.661, 0.999, and 0.999, and the IgM correlations are 0.784, 0.933, 0.892, and 0.956, respectively. To further confirm these findings, we analyzed all the samples (Supplementary Table S1, Cohort 2) of the 5 time points (Fig. 1e–g), and the correlations between WT and Omicron for IgG, IgA, and IgM are 0.995, 0.409, and 0.852, respectively. These results indicate that the epitopes on RBD that the antibodies bind to are randomly distributed; though antibody responses against RBD-Omicron are lower than those against RBD-WT, the ratios are relatively consistent during the whole vaccination process. Further, we compared the consistence among IgG, IgM, and IgA, for RBD-WT, the correlations of IgG/IgM, IgG/IgA, and IgM/IgA are –0.450, 0.921, and –0.408, respectively; for RBD-Omicron, the correlations of IgG/IgM, IgG/IgA, and IgM/IgA are –0.374, 0.321, and 0.705, respectively. Overall, the IgG and IgM responses are negatively consistent, which is not unexpected according to the different time points that IgG and IgM are generated after infection or immunization. Interestingly, we observed positive correlations between IgG and IgA, which suggests that the inactivated virus vaccine can also trigger mucosal immunity, and it can last a similar time frame to the IgG response.

Importantly, we provide the first data from people who were vaccinated with the inactivated virus, revealing significantly lower antibody binding to the Omicron versus WT RBD at all stages of the vaccination process. We found that, while there is clearly some protection against the Omicron variant, it is only after the booster shot that the immune responses of both IgG and IgA are as efficient for the Omicron as those after the second shot for the WT^9,10^. Thus, while the booster may be useful to combat the WT strain, it might be ultimately essential to neutralize the Omicron variant.

Our study has some limitations. The accessibility to a biosafety facility is very limited, and thus we have not confirmed our results with the authentic WT virus and Omicron variant. However, because of the positive correlation between RBD binding and neutralization activity towards the authentic virus, the flexible microarray platform would be suitable for rapid evaluation in the future. When facing new variant of concern (VOC), the microarray could be easily upgraded by including the RBD of the new VOC. To make full use of this microarray, it could be further developed for routine application by incorporating visual detection.

In addition, the cohort is small. However, to reduce the person-to-person variation, we collected longitudinal samples during the last year. Still, our findings could be strengthened by a larger cohort. Due to the availability, we only analyzed samples collected from people who were vaccinated with the inactivated virus vaccine BBIBP-CorV. It will also be interesting to analyze longitudinal samples of other type of vaccines.

In summary, our results provide important insights into the antibody protection against the Omicron variant throughout the vaccination stages. Our results strongly support the necessity of booster vaccination. Post-booster vaccination may also need to be considered.

## Supplementary information

Supplementary information

## Data Availability

The SARS-CoV-2 RBD protein microarray data are deposited to Protein Microarray Database under the accession number PMDE260 (http://www.proteinmicroarray.cn/). Additional data related to this paper are available from the corresponding author upon reasonable request.

## References

[1] Cele, S. et al. SARS-CoV-2 Omicron has extensive but incomplete escape of Pfizer BNT162b2 elicited neutralization and requires ACE2 for infection. *medRxiv*10.1101/2021.12.08.212674177 (2021).

[2] Dejnirattisai, W. et al. SARS-CoV-2 Omicron-B.1.1.529 leads to widespread escape from neutralizing antibody responses. *Cell*10.1016/j.cell.2021.12.046 (2022). 10.1016/j.cell.2021.12.046 PMC8723827 35081335

[3] World Health Organization. WHO Coronavirus (COVID-19) Dashboard https://covid19.who.int (2021).

[4] Ma ML (2021). Systematic profiling of SARS-CoV-2-specific IgG responses elicited by an inactivated virus vaccine identifies peptides and proteins for predicting vaccination efficacy. Cell Discov..

[5] Planas, D. et al. Considerable escape of SARS-CoV-2 Omicron to antibody neutralization. *Nature*10.1038/s41586-021-04389-z (2021). 10.1038/s41586-021-04389-z 35016199

[6] Sievers, B. L. et al. Antibodies elicited by SARS-CoV-2 infection or mRNA vaccines have reduced neutralizing activity against Beta and Omicron pseudoviruses. *Sci. Transl. Med.*10.1126/scitranslmed.abn7842 (2022). 10.1126/scitranslmed.abn7842 PMC8891085 35025672

[7] Hand TW, Reboldi A (2021). Production and function of immunoglobulin A. Annu. Rev. Immunol..

[8] Sterlin D (2021). IgA dominates the early neutralizing antibody response to SARS-CoV-2. Sci. Transl. Med..

[9] Ai J (2022). Omicron variant showed lower neutralizing sensitivity than other SARS-CoV-2 variants to immune sera elicited by vaccines after boost. Emerg. Microbes Infect..

[10] Yu, X. et al. Reduced sensitivity of SARS-CoV-2 Omicron variant to booster-enhanced neutralization. *medRxiv*10.1101/2021.12.17.21267961 (2021).

