# Supplementary material for: Landscape of the RBD-specific IgG, IgM, and IgA responses triggered by the inactivated virus vaccine against the Omicron variant
Source: Cell Discov. 2022 Feb 15;8:15. doi: 10.1038/s41421-022-00380-8 (PMC8847627; doi:10.1038/s41421-022-00380-8)
Supplement: Supplementary file 1 — Supplementary information [file 41421_2022_380_MOESM1_ESM.pdf]

# Landscape of the RBD-specific IgG, IgM and IgA responses triggered by the inactivated virus vaccine against the Omicron variant

Jun-biao Xue[1, #], Dan-yun Lai[1, #], He-wei Jiang [1, #], Huan Qi[1], Shu-Juan Guo[1], Yuan-shou Zhu[1], Hong Xu [2], Jie Zhou[3], Sheng-ce Tao[1, *]

[1]Key Laboratory of Systems Biomedicine (Ministry of Education), Shanghai Center for Systems Biomedicine, Shanghai Jiao Tong University, Shanghai 200240, China

[2]School of Biomedical Engineering, Shanghai Jiao Tong University, Shanghai 200240, China

[3]Foshan Fourth People's Hospital, Foshan 528000, China

[#]These authors contributed equally to this study

[*]Correspondence: taosc@sjtu.edu.cn (S.-c. T.)

## Methods

### Protein microarray fabrication

The RBD proteins (Acro Biosystem, Beijing, China) and S1 protein (Abclonal, Wuhan, China) of SARS-CoV-2, along with the negative (BSA) and positive controls (Human IgG and IgM), were printed in five replicates on PATH substrate slide (Grace Bio-Labs, Oregon, USA) to generate identical arrays in a 2 x 7 subarray format using Super Marathon printer (Arrayjet, UK). The microarrays were stored at -80°C until use.

### Patients and samples

All the RBD-specific mAbs were provided by Sanyou Bioparmaceuticals (Shanghai, China). A total of 14 COVID-19 convalescent patients and 13 participants of vaccination were enrolled in this study. The patients were hospitalized and received treatment in Foshan Forth hospital, the serum samples were collected on the day of hospital discharge (Cohort 1 in **Table S1**). A total of 13 participants of vaccination were recruited from the beginning of 2021 (Cohort 2 in **Table S1**), the participants were vaccinated with the inactivated virus vaccine BBIBP-CorV, and longitudinal serum samples were collected at 16 time points during one year, the samples included unvaccinated, the $1^{st}$ dose, the $2^{nd}$ dose and the $3^{rd}$ (booster) dose (**Figure 1A**).

### Microarray-based serum and antibody analysis

A 14-chamber rubber gasket was mounted onto each slide to create individual chambers for the 14 identical subarrays. The microarray was used for serum profiling as described previously with minor modifications[1]. Briefly, the arrays were brought from -80°C to -20°C and room temperature for gradient rewarming and then

incubated in blocking buffer (3% BSA in 1×PBS buffer with 0.1% Tween 20) for 3 h. A total of 200 μL of diluted sera was incubated with each subarray overnight at 4°C, monoclonal antibodies was incubated 2h at RT. The sera were diluted at 1:200. The antibody was diluted to a final concentration of 10 μg/mL for incubation. The microarrays were washed with 1×PBST and the bound antibodies were detected by incubating with Cy3-conjugated goat anti-human IgG and Alexa Fluor 647-conjugated donkey anti-human IgM (Jackson ImmunoResearch, PA, USA), which were diluted for 1:1,000 in 1×PBST. The incubation was carried out at room temperature for 1 h. The microarrays were then washed with 1×PBST and dried by centrifugation at room temperature. The microarrays were scanned by LuxScan 10K-A (CapitalBio Corporation, Beijing, China) with the parameters set as 100% laser power/ PMT 550, 100% laser power/ PMT 500 for IgM and IgG (Cy5 and Cy3 channel), respectively. For IgA (Fluorescein channel), the microarrays were scanned by GenePix 4200A (Molecular Devices, CA, USA) with the parameters set as 100% laser power/PMT 400. The fluorescent intensity was extracted by GenePix Pro 6.0 software (Molecular Devices, CA, USA).

**Ethical approval**

The study was approved by the Institutional Ethics Review Committee of Foshan Fourth Hospital, Foshan, China (ref. no. 202005) and the Ethics Commission of Shanghai Jiao Tong University (ref. no. B2021120I). The written informed consent was obtained from each participant. Participants of vaccination have been informed the sera collection of post-booster vaccination and the written informed consent was obtained from each participant.

**Data analysis of protein microarray**

For each spot, signal intensity was defined as the median_foreground subtracted by the median_background. The signal intensities of the five replicates spots for each peptide or protein were averaged. IgG, IgM and IgA data were analyzed separately. *P-values* for statistical analysis were calculated by unpaired two-way *t*-test. Correlations were calculated by pairwise analysis. All diagram and statistical analyses were carried out using GraphPad Prism 8 and IBM SPSS Statistics 24.

## Supplementary References


1 Ai, J. *et al.* Omicron variant showed lower neutralizing sensitivity than other SARS-CoV-2 variants to immune sera elicited by vaccines after boost. *Emerg. Microbes Infect.*, 1-24, doi:10.1080/22221751.2021.2022440 (2021).

## Figure legends

**Fig. S1 The Omicron RBD protein microarray. a** The layout of the protein microarray; **b** Quality inspection of the protein microarray which was probed with anti-6xHis, anti-Human_IgG and anti-Human_IgM antibodies; **c-d** The bindings of RBD-specific monoclonal antibodies on the microarray (**c**) and 14 COVID-19 convalescent sera (**d**) against RBD-WT (wild type) and RBD-Omicron. The black horizontal lines indicate the average signal intensity. Numbers in black (**d**) denote fold-change in signal intensity between RBD-WT and RBD-Omicron.

**Fig. S2 RBD-specific IgG antibody responses of longitudinal sera collected from vaccinated individuals. a-c** The trends of RBD-specific antibody responses during one year for 3 individuals that were immunized with 3 doses of inactivated virus vaccine, Donor002(**a**), Donor003(**b**) and Donor004(**c**).

**Fig. S3 RBD-specific IgA responses of longitudinal sera collected from vaccinated individuals. a-c**, The trends of RBD-specific IgA responses during one year for 3 individuals that were immunized with 3 doses of inactivated virus vaccine, Donor002(**a**), Donor003(**b**) and Donor004(**c**). **d-e** The IgA responses against RBD-WT and RBD-Omicron at 2-2 (**d**) and 3-2 (**e**).

**Fig. S4 RBD-specific IgM responses of longitudinal sera collected from vaccinated individuals. a-c** The trends of RBD-specific IgM responses during one year for 3 individuals that were immunized with 3 doses of inactivated virus vaccine, Donor002(**a**), Donor003(**b**) and Donor004(**c**). **d-e** The IgM responses between RBD-

WT and RBD-Omicron at 2-2 (**d**) and 3-2 (**e**). **f-g** The IgM responses at 2-2 and 3-2 against RBD-WT (**f**) and RBD-Omicron (**g**). The trends of Omicron-RBD specific IgM responses after the 3$^{rd}$ dose (**h**).

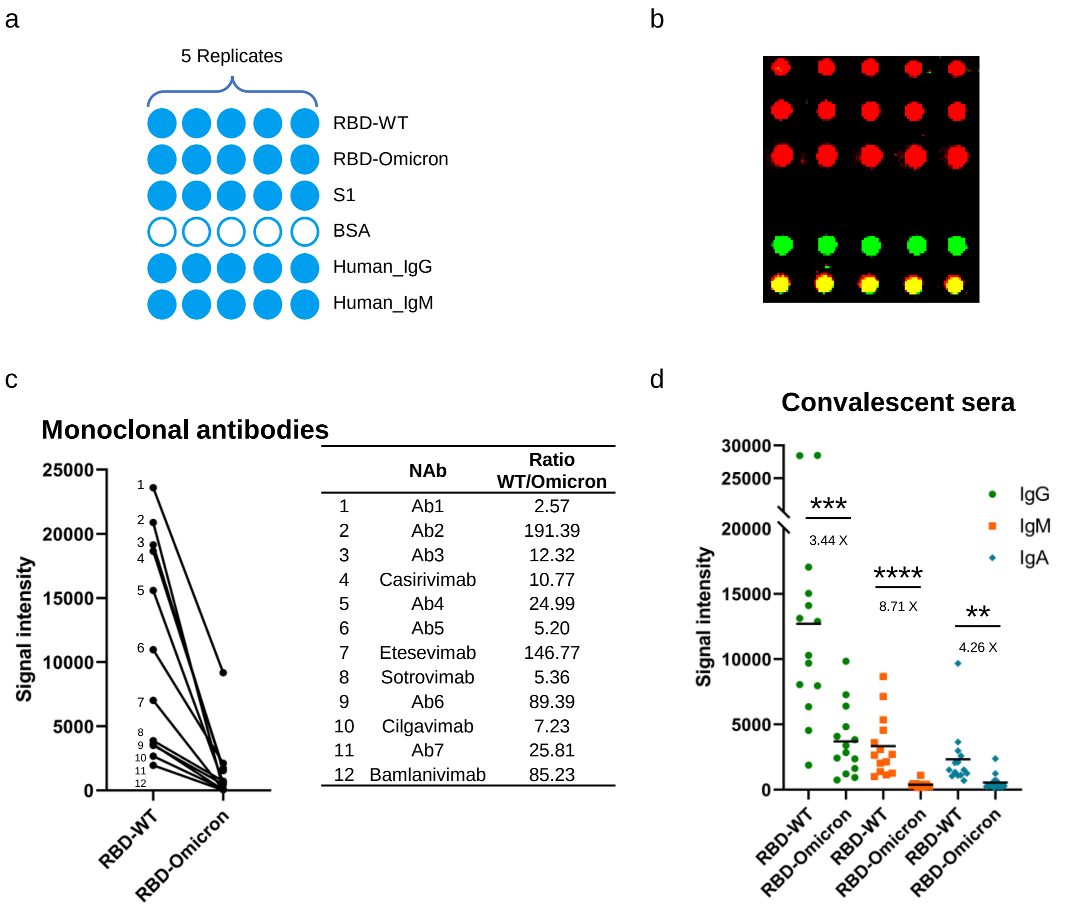


| | NAb | Ratio WT/Omicron |
|---|---|---|
| 1 | Ab1 | 2.57 |
| 2 | Ab2 | 191.39 |
| 3 | Ab3 | 12.32 |
| 4 | Casirivimab | 10.77 |
| 5 | Ab4 | 24.99 |
| 6 | Ab5 | 5.20 |
| 7 | Etesevimab | 146.77 |
| 8 | Sotrovimab | 5.36 |
| 9 | Ab6 | 89.39 |
| 10 | Cilgavimab | 7.23 |
| 11 | Ab7 | 25.81 |
| 12 | Bamlanivimab | 85.23 |

**Fig. S1 The Omicron RBD protein microarray.**

a

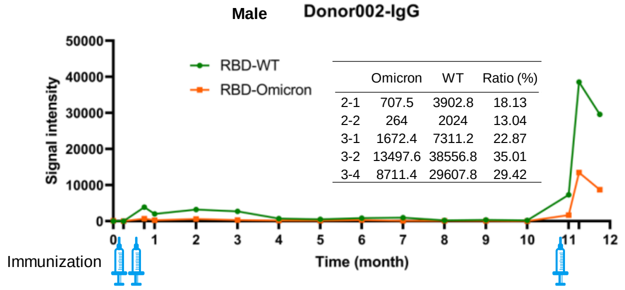


b

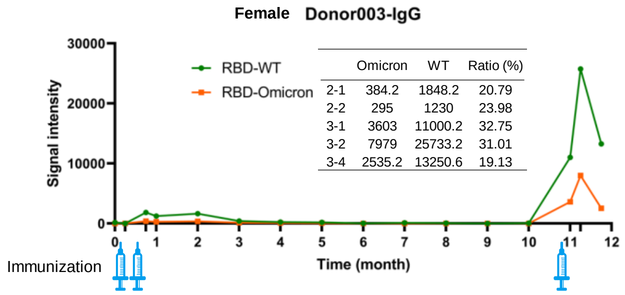


c

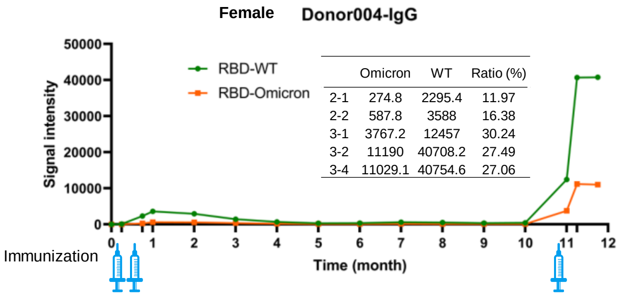


**Fig. S2 RBD-specific IgG responses of longitudinal sera collected from vaccinated individuals.**

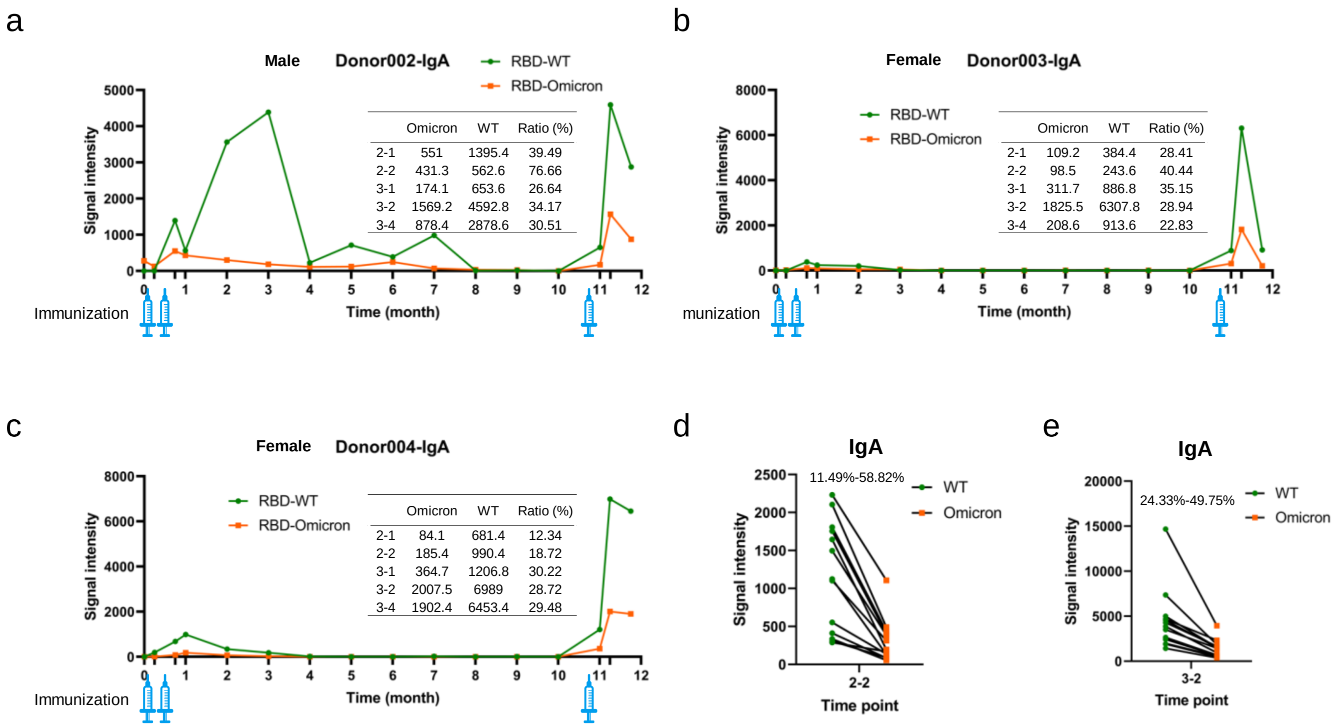


**Fig. S3 RBD-specific IgA responses of longitudinal sera collected from vaccinated individuals.**

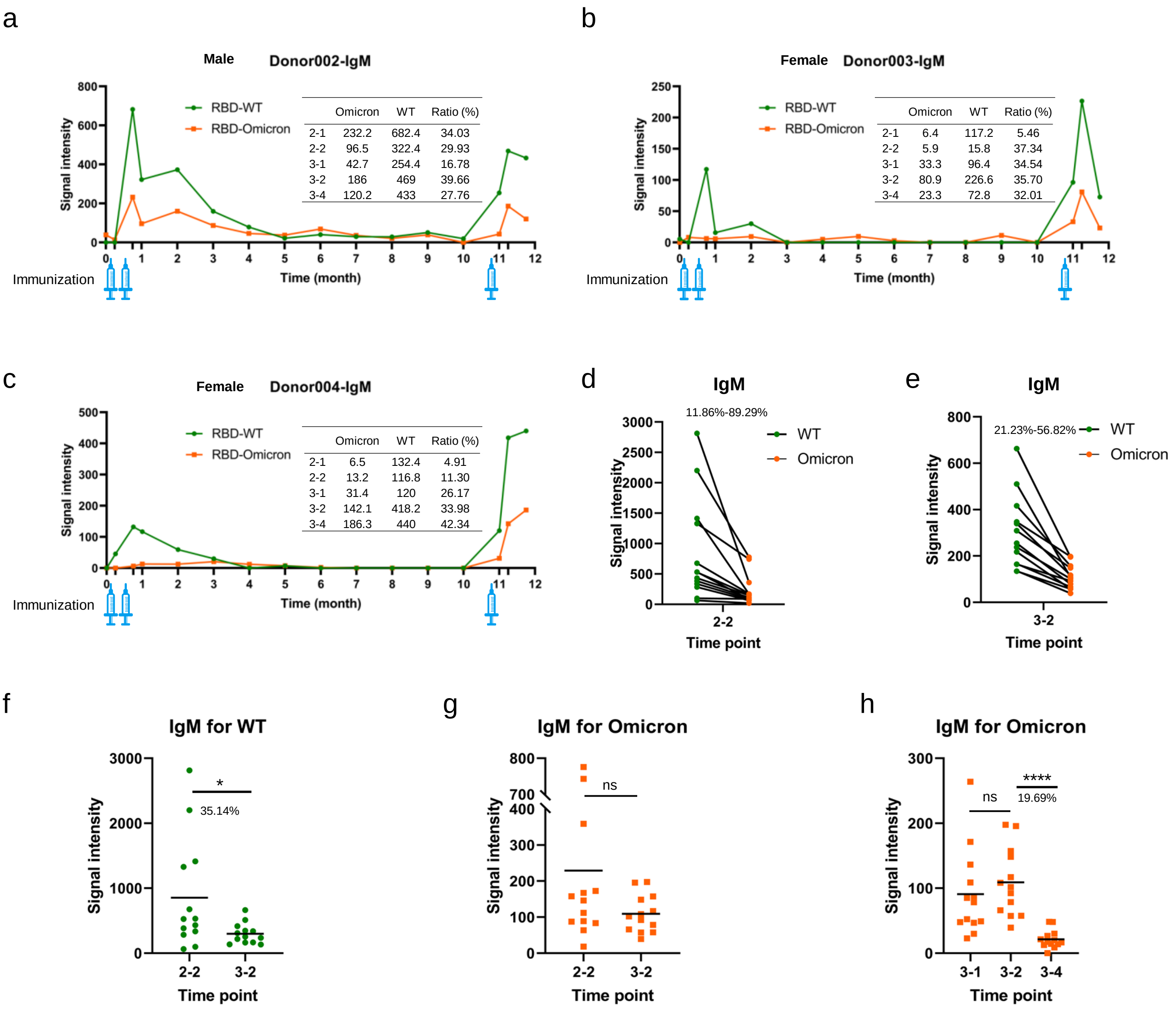


**Fig. S4 RBD-specific IgM responses of longitudinal sera collected from vaccinated individuals.**

## Table S1 Information of samples used in this study.

| | | Cohort 1 | Cohort 2 |
|---|---|---|---|
| **Group** | | **Convalescent** | **Vaccination** |
| **Numbers** | | 14 | 13 |
| **Age** | | 44.7±15.5 | 40.2±7.9 |
| **Gender** | Male | 7 | 11 |
| | Female | 7 | 2 |
| **Severity** | Severe | 0 | - |
| | non-severe | 14 | |
| | Death | 0 | |
| **Days after onset** | | 25.6±4.9 | - |
| **Source** | | Foshan 4th Hospital, Guangdong | Shanghai Jiao Tong University, Shanghai |
